# Therapeutic Applications and Target Strategies of Antibody-Drug Conjugates in Ovarian Cancer

**DOI:** 10.5812/ijpr-165383

**Published:** 2025-11-08

**Authors:** Qiming Liu, Runji Zhou, Taiqi He, Yonghong Zhao, Mei Yang

**Affiliations:** 1 School of Basic Medicine, Southwest Medical University, Luzhou, China; 2 Department of Clinical Medicine, Xinjiang Medical University, Urumqi, China; 3 Department of Gynecology and Obstetrics, Santai County People’s Hospital, Mianyang, China

**Keywords:** Antibody-Drug Conjugates (ADCs), Ovarian Cancer, Targeted Therapy, Clinical Trials

## Abstract

**Context:**

Ovarian cancer remains a significant global health concern, characterized by limited therapeutic options and high mortality rates, primarily due to late-stage diagnosis and resistance to conventional therapies. Antibody-drug conjugates (ADCs) represent a promising targeted therapeutic approach for this malignancy.

**Evidence Acquisition:**

A comprehensive literature search was conducted using PubMed/MEDLINE, Scopus, Web of Science, and Google Scholar databases. Studies were selected based on their relevance to the development of ADCs, as well as their preclinical and clinical evaluation in ovarian cancer, resistance mechanisms, and toxicity profiles.

**Results:**

This review summarizes a range of ADCs targeting tumor-associated antigens in ovarian cancer, including mirvetuximab soravtansine (MIRV), trastuzumab deruxtecan (T-DXd), datopotamab deruxtecan (Dato-DXd), sacituzumab tirumotecan (SKB-264), PF-06664178, anetumab ravtansine (BAY 94-9343), BMS-986148, DMOT4039A, RC88, lifastuzumab vedotin (DNIB0600A), upifitamab rilsodotin (ABBV-181), ZW220, DMUC4064A, and sofituzumab vedotin (DMUC5754A). Resistance mechanisms, toxicity profiles, and the potential for combination therapies and next-generation ADC designs are also discussed.

**Conclusions:**

The ADCs hold significant potential to reshape the treatment landscape for ovarian cancer by providing targeted therapeutic options. Further research is required to optimize patient selection, address resistance mechanisms, and improve safety profiles.

## 1. Context

Ovarian cancer is the eighth most common cancer in women worldwide and the second most common gynecologic malignancy after endometrial cancer. In 2022, approximately 325,000 new cases and 207,000 deaths occurred globally, with an age-standardized incidence rate of 6.7 and a mortality rate of 4.0 per 100,000 women. Incidence is highest in Europe, North America, Australia, and New Zealand, primarily affecting postmenopausal women aged fifty-five to seventy years, with 70% diagnosed at advanced stages (1). By 2050, new cases are projected to exceed 500,000, and deaths may rise to more than 350,000 annually, driven by demographic shifts and aging populations. In Iran, ovarian cancer is the eighth most common cancer among women, with an incidence rate of 4.1 per 100,000 and about 2,000 new cases annually. Data from 2009 to 2014 show 7,977 cases, with five- and ten-year survival rates of 55% and 44%, respectively, reflecting regional epidemiology influenced by declining fertility and an aging population (2, 3).

This stark reality underscores the pressing need for innovative therapeutic strategies that can improve outcomes for patients facing this formidable disease. Conventional treatments for ovarian cancer, such as surgery and chemotherapy, often fall short due to challenges such as tumor heterogeneity, resistance to conventional therapies, and significant side effects (4). As a result, there is a growing emphasis on targeted therapy, which aims to selectively attack cancer cells while sparing healthy tissues (5). Targeted therapies have shown promise in enhancing treatment efficacy and minimizing adverse effects, thus representing a critical advancement in the management of ovarian cancer (6).

**Figure 1.**
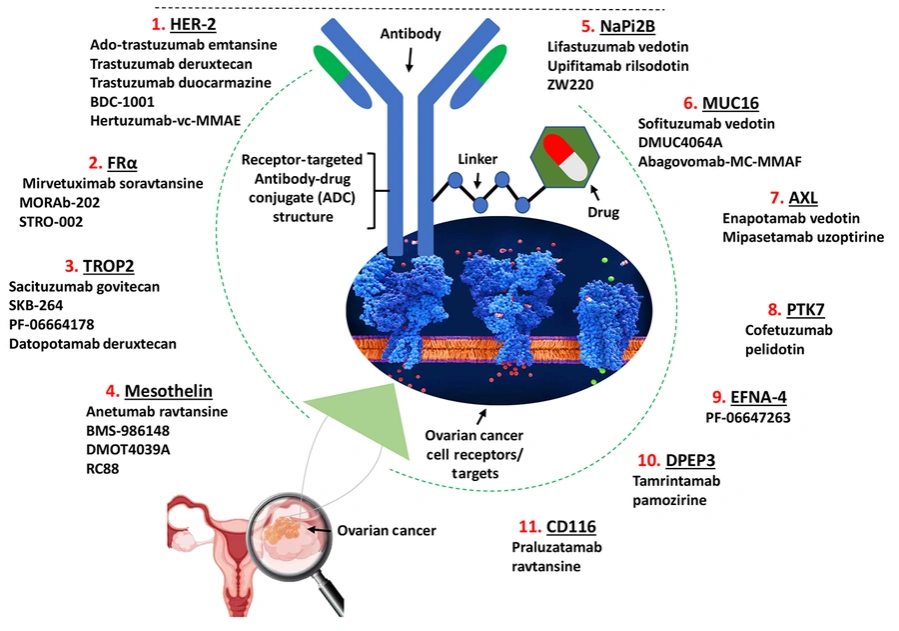
Mechanism of antibody-drug conjugates (ADCs) targeting ovarian cancer cells – the ADC structure is shown binding to these receptors (numbers 1 - 11 indicate ADCs targeting specific antigens).

Among the most promising advancements in targeted therapy are antibody-drug conjugates (ADCs). These novel therapeutic agents combine the specificity of monoclonal antibodies with the potency of cytotoxic drugs, facilitating targeted delivery directly to tumor cells (7, 8). The ADCs act through a mechanism that allows them to bind to specific tumor-associated antigens, delivering their cytotoxic payload directly into cancer cells while largely sparing surrounding healthy tissue (Figure 1) (9). This targeted approach not only enhances therapeutic efficacy but also reduces systemic toxicity, making ADCs an attractive option for patients with recurrent or resistant ovarian cancer (10, 11).

This review article explores the emerging applications of ADCs in targeted therapy for ovarian cancer. The discussion will begin with an overview of ADCs, detailing their structure and function, followed by an examination of their emerging applications specifically within the context of ovarian cancer treatment. Specific examples of ADCs currently being evaluated in clinical trials will be highlighted, showcasing their potential impact on patient outcomes. Additionally, we will address the challenges and limitations of ADCs in ovarian cancer therapy, including issues related to drug resistance and patient selection.

## 2. Evidence Acquisition

### 2.1. Literature Search Strategy and Study Selection Criteria

The literature search was conducted using multiple electronic databases — PubMed/MEDLINE, Scopus, Web of Science, and Google Scholar — employing search terms such as “Antibody-Drug Conjugate”, “Ovarian Cancer”, specific ADC names, and target antigens. Supplementary sources included conference proceedings and regulatory websites to ensure comprehensive coverage of recent advances. Studies published from January 1, 2010, to December 31, 2024, were included to capture the full evolution of ADC development in ovarian cancer, from early preclinical reports to key clinical advancements. Data extraction focused on study characteristics, ADC properties, preclinical and clinical outcomes, including response rates, survival data, and toxicity profiles. Studies lacking ovarian cancer-specific ADC data or published outside this timeframe were excluded.

### 2.2. Inclusion Criteria and Study Grouping

This review comprehensively includes all reported ADCs targeting ovarian cancer antigens from both preclinical and clinical studies. Inclusion criteria encompassed original studies reporting ADC targets, therapeutic payloads, clinical trial outcomes, and molecular characteristics. Studies without specific ADC data or unrelated to ovarian cancer were excluded. Data were synthesized by grouping ADCs according to their target antigens, with key characteristics summarized in tables presenting antigen features (Table 1), clinical trial statuses and outcomes (Table 2), and ADC molecular designs (Table 3). 

**Table 1.** Characteristics of Potential Targets for Antibody-Drug Conjugates in Ovarian Cancer

Targets	Antigen ID	Family	Function	KEGG ID	Expression in Ovarian Cancer Tissue ^a^ (Compared with the Healthy Tissue)
**HER-2**	TAR0THKZD	Tyr protein family	Regulates outgrowth and stabilization of peripheral microtubules	hsa: 2064	P-value: 0.000490484; fold-change: 0.452221886; Z-score: 1.023085171
**FRα**	TAR0QHAVI	Ephrin family	Mediates delivery of 5-methyltetrahydrofolate and folate into the interior of cells; needed for normal cell proliferation	hsa: 2348	P-value: 0.000299394; fold-change: 1.814366231; Z-score: 2.811538221
**TROP2**	TAR0MIBZW	EPCAM family	Growth factor receptor	hsa: 4070	P-value: 0.599278238; fold-change: 0.03145742; Z-score: 0.093963239
**Mesothelin**	TAR0NEZUA	LAMP family	Cellular adhesion	hsa: 10232	P-value: 0.013548348; fold-change: 5.443607931; Z-score: 1.915568501
**NaPi2B**	TAR0AABNG	SEZ6 family	Transporting phosphate into cells via Na(+) cotransport	hsa: 10568	P-value: 0.000252513; fold-change: 3.585892004; Z-score: 2.614444631
**MUC16**	TAR0WGRJX	Mucin family	A protective, lubricating barrier at mucosal surfaces	hsa: 94025	P-value: 0.000485959; fold-change: 5.023844415; Z-score: 2.60194028
**AXL**	TAR0PMKUR	Tyr protein family	Transduces signals from the ECM into the cytoplasm	hsa: 558	P-value: 0.002328813; fold-change: -0.429297563; Z-score: -1.07444801
**PTK7**	TAR0JXPTV	Tyr protein family	Cell adhesion, cell migration, cell polarity, proliferation, actin cytoskeleton reorganization and apoptosis	hsa: 5754	P-value: 0.217792723; fold-change: -0.156908615; Z-score: -0.325247462
**EFNA-4**	TAR0IMOZN	EPCAM family	Migration, repulsion and adhesion during neuronal, vascular and epithelial development	hsa: 1945	P-value: 0.002363649; fold-change: 0.538766502; Z-score: 1.102294908
**DPEP3**	TAR0XXQUD	Mesothelin family	Tumor biology, hydrolysis of dipeptides	hsa: 64180	P-value: 0.000223723; fold-change: 0.053522424; Z-score: 0.160746042
**CD116**	TAR0UPRHI	immunoglobulin superfamily	T-cell activation and proliferation, formation and maturation of the immunological synapse	hsa: 214	P-value: 0.183621643; fold-change: -0.350627328; Z-score: -0.487609969

Abbreviations: FRα, folate receptor alpha; TROP2, trophoblast cell surface antigen 2.

^a^ Data source: ADCdb, https://adcdb.idrblab.net.

**Table 2.** Summary of Antibody-Drug Conjugates in Clinical Trials for Ovarian Cancer

ADC	Selected Clinical Trials No.	Clinical Status	Therapeutic Target	TGI (%)	ORR (%)
**Ado-trastuzumab emtansine**	NCT01702571	II/III/1b	Microtubule	45	21.40
**T-DXd**	NCT02564900	I	DNA topoisomerase 1	88 - 96	37
**Trastuzumab duocarmazine**	NCT04235101	I	hDNA	33 - 66	27.8
**BDC-1001**	NCT04278144	I/II	TLR7/8	29	33 - 37
**Hertuzumab-vc-MMAE**	NCT02001623	I/II	Microtubule	61 - 100	13.89
**MIRV**	NCT04296890	III/1b/2	Microtubule	33 - 100	22 - 39
**MORAb-202**	NCT03386942	I	Microtubule	2 - 98	31 - 50
**STRO-002**	NCT03748186	I	Microtubule	15 - 100	33 - 47
**Sacituzumab govitecan**	NCT01631552	I/II	DNA topoisomerase 1	37 - 97	32 - 35
**SKB-264**	NCT05631262; NCT04152499	I/II	DNA topoisomerase 1	30 - 100	40 - 60
**PF-06664178**	NCT02122146	I	Microtubule	-	37.9
**Dato-DXd**	NCT05489211	II	DNA topoisomerase 1	85.7	42.9
**BAY 94-9343**	NCT03587311	II	Microtubule	42 - 100	27.70
**BMS-986148**	NCT02341625	I/IIa	Microtubule	100	2 - 23
**DMOT4039A**	NCT03657043	I/II	Microtubule	72 - 100	27
**RC88**	NCT03657043	I/II	Microtubule	95 - 100	13 - 40
**DNIB0600A**	NCT01991210	I/Ib/II	Microtubule	41.2	25 - 75
**ABBV-181**	NCT05329545	I/II/III	Microtubule	95	29 - 34
**ZW220**	Preclinical	-	DNA topoisomerase 1	-	-
**DMUC5754A**	NCT03657043	II	Microtubule	72 - 100	13 - 40
**DMUC4064A**	NCT02146313	I	Microtubule	-	35 - 45
**Abagovomab-MC-MMAF**	MIMOSA trial	III	Microtubule	-	-
**Enapotamab vedotin**	NCT03245736	II	Microtubule	72 - 99	40
**Mipasetamab uzoptirine**	NCT05389462	II	hDNA	34 - 98	23 - 47
**Cofetuzumab pelidotin**	NCT03245736	II	Microtubule	72 - 100	40
**PF-06647263**	NCT02078752	I	hDNA	100	36
**Tarangambadi pamozirine**	NCT02539719	I	hDNA	100	5.17
**Praluzatamab ravtansine**	NCT03149549	I/II	Microtubule	100	9

Abbreviations: ADC, antibody-drug conjugate; TGI, tumor growth inhibition value; ORR, objective response rate; T-DXd, trastuzumab deruxtecan; hDNA, human deoxyribonucleic acid; TLR7/8, toll-like receptors 7 and 8; MMAE, monomethyl auristatin E; MIRV, mirvetuximab; SKB-264, sacituzumab tirumotecan; Dato-DXd, soravtansine; datopotamab deruxtecan; MMAFD, monomethyl auristatin F.

**Table 3.** Composition and Design of Antibody-Drug Conjugates Targeting Ovarian Cancer Antigens

Antigen and ADCs	ADC ID	Payload	Linker	Antibody
**HER-2**				
Ado-trastuzumab emtansine	DRG0CYMEB	Mertansine DM1	SMCC (ID: LIN0EBJON); flexible dual-reactive (amino/thiol) linker	Trastuzumab
T-DXd	DRG0ERKBH	Deruxtecan (DX-8951 derivative)	Mc-Gly-Gly-Phe-Gly (ID: LIN0ECMCR); cathepsin-cleavable linker	Trastuzumab
Trastuzumab duocarmazine	DRG0THGAW	Seco-DUBA	Mal-PEG2-Val-Cit-PABA-Cyclization Spacer (ID: LIN0HGTZQ); cathepsin-cleavable linker	Trastuzumab
BDC-1001	DRG0HVSBG	TORL7/8 agonist T785	BG based linker (LIN0SXPBU); bioorthogonal reaction linker	Trastuzumab
Hertuzumab-vc-MMAE	DRG0TCBEP	MMAE	Mc-Val-Cit-PABC (ID: LIN0SQEDQ); cathepsin-cleavable linker	Hertuzumab
**FRα**				
MIRV	DRG0GKOZH	Mertansine DM4	Sulfo-SPDB (ID: LIN0MESCO); flexible dual-reactive (amino/thiol) linker (thiol-sensitive linker)	Mirvetuximab
MORAb-202 (farletuzumab ecteribulin)	DRG0HPUIL	Eribulin	Mal-PEG2Val-Cit-PAB-OH (ID: LIN0KCWDR); cathepsin-cleavable linker	Farletuzumab
STRO-002 (luveltamab tazevibulin)	DRG0RRDEF	Hemiasterlin-derivative	Val-Cit-PABA (ID: LIN0CSOSO); cathepsin-cleavable linker	Luveltamab
**TROP2**				
Sacituzumab govitecan	DRG0EKTUN	Active metabolite of irinotecan SN38	CL2A (ID: LIN0ZGCUP); pH-sensitive linker	Sacituzumab
SKB-264	DRG0BSQPI	KL610023	Pyrimidine-CL2A-carbonate (ID: LIN0ZEYRW); pH-sensitive linker	Sacituzumab
PF-06664178	DRG0KTRKE	Auristatin-0101	AcLys-Val-Cit-PABC (ID: LIN0FSRSR); cathepsin-cleavable linker	PF-06478924
Dato-DXd	DRG0ZOYQV	DX-8951 derivative (DXd)	Mc-Gly-Gly-Phe-Gly (ID: LIN0ECMCR); cathepsin-cleavable linker	Datopotamab
**Mesothelin**				
BAY 94-9343	DRG0EPHMC	Mertansine DM4	SPDB (ID: LIN0VZYER); thiol-sensitive linker	Anetumab
BMS-986148	DRG0VYGGK	Tubulysin	Mal-EBE-Mal (ID: LIN0ATYUQ); thiol-sensitive linker	BMS-986021
DMOT4039A	DRG0FXFDD	MMAE	Mc-Val-Cit-PABC (ID: LIN0SQEDQ); cathepsin-cleavable linker	AMA(MMOT0530A)
RC88	DRG0DVPFF	MMAE	Mc-Val-Cit-PABC (ID: LIN0SQEDQ); cathepsin-cleavable linker	Anti-MSLN mAb
**NaPi2B**				
DNIB0600A	DRG0UQTJG	MMAE	Mc-Val-Cit-PABC (ID: LIN0SQEDQ); cathepsin-cleavable linker	Lifastuzumab
ABBV-181	DRG0OZSZX	Auristatin F hydroxypropylamide (AF-HPA)	Dolaflexin polymer (ID: LIN0SWMHT); polymer linker	XMT-1535
ZW220	DRG0CFNKG	ZD06519	Mc-Gly-Gly-Phe-Gly-AM (ID: LIN0YIZVG); cathepsin-cleavable linker	Fully humanized anti-SLC34A2 IgG1 mAb
**MUC16**				
DMUC5754A	DRG0RSBMJ	MMAE	Mc-Val-Cit-PABC (ID: LIN0SQEDQ); cathepsin-cleavable linker	Anti-MUC16 antibody 3A5
DMUC4064A	DRG0NKVYN	MMAE	Protease cleavable linker (ID: LIN0RNDLI)	MMUC3333A
Abagovomab-MC-MMAF	ADC-W-1636	MMAF	MC (maleimidocaproyl)	Abagovomab
**AXL**				
Enapotamab vedotin	DRG0MCWQA	MMAE	Mc-Val-Cit-PABC (ID: LIN0SQEDQ); cathepsin-cleavable linker	AXL-107
Mipasetamab uzoptirine	DRG0TZZNJ	SG3199	BCN-HydraSpace-Val-Ala-PABC (ID: LIN0WLMUR); cathepsin-cleavable linker	Mipasetamab
**PTK7**				
Cofetuzumab pelidotin	DRG0XHZLG	Auristatin-0101	Mc-Val-Cit-PABC (ID: LIN0SQEDQ); cathepsin-cleavable linker	Cofetuzumab
**EFNA-4**				
PF-06647263	DRG0SHGPV	N-acetyl-gamma-calicheamicin	AcButDMH (ID: LIN0KWKDL); pH-sensitive linker	Anti-EFNA4 mAb huE22
**DPEP3**				
Tamrintamab pamozirine	DRG0RZJIL	SC-DR002	Mc-Val-Cit-PABC (ID: LIN0SQEDQ); cathepsin-cleavable linker	Tamrintamab
**CD116**				
Praluzatamab ravtansine	DRG0QTTWI	Mertansine DM4	N-succinimidyl 4-(2-pyridyldithio) butanoate (ID: LIN0VZYER); thiol-sensitive linker	Praluzatamab

Abbreviations: ADC, antibody-drug conjugate; T-DXd, trastuzumab deruxtecan; MMAE, monomethyl auristatin E; FRα, folate receptor alpha; MIRV, mirvetuximab soravtansine; TROP2, trophoblast cell surface antigen 2; SKB-264, sacituzumab tirumotecan; DatO-DXd, datopotamab deruxtecan; MMAF, monomethyl auristatin F.

### 2.3. An Overview of Antibody-Drug Conjugates

#### 2.3.1. Mechanism of Antibody-Drug Conjugates

**Figure 2.**
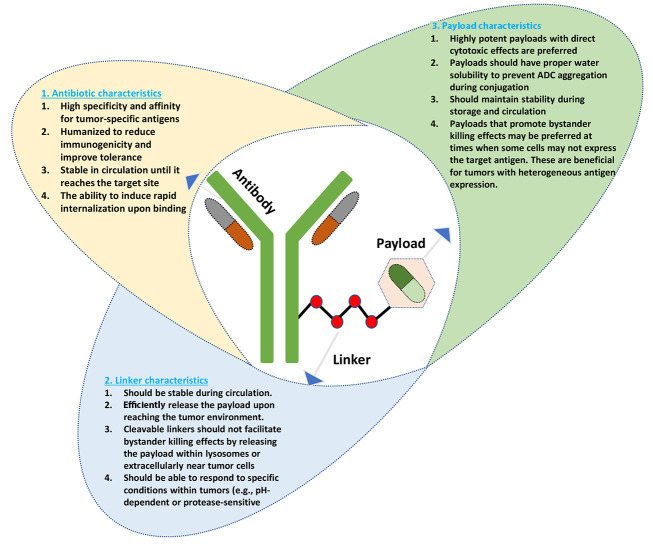
Components and characteristics of antibody-drug conjugates (ADCs); schematic representation of an ADC structure, highlighting its three main components: A, antibody; B, linker; and C, payload (arrows indicate the characteristics associated with each component).

The ADCs are a novel class of biopharmaceuticals designed to enhance the precision and efficacy of cancer therapy (12). Consisting of a monoclonal antibody linked to a cytotoxic drug via a stable chemical linker, ADCs leverage the specific targeting capabilities of antibodies to deliver potent anti-cancer agents directly to tumor cells (13). The mechanism of action involves several key steps: First, the antibody component binds to specific antigens expressed on the surface of cancer cells, facilitating internalization of the entire conjugate into the cell. Once inside, the linker is cleaved — either through enzymatic degradation or changes in the cellular environment — releasing the cytotoxic payload. This released drug then exerts its cytotoxic effects, often by damaging DNA or disrupting microtubule function, which ultimately leads to cancer cell death. This targeted approach minimizes damage to surrounding healthy tissues, a significant advantage compared to traditional chemotherapy (14, 15). The components and characteristics of ADCs (Figure 2) are illustrated to demonstrate the structural basis of ADC-based therapy for ovarian cancer.

#### 2.3.2. Advantages of Antibody-Drug Conjugates

The advantages of ADCs in targeted cancer therapy are manifold. A principal benefit is their ability to selectively target cancer cells while sparing normal cells, which significantly reduces systemic toxicity and improves patient tolerability (16). This selectivity enables higher doses of cytotoxic agents to be delivered directly to tumors without the common side effects associated with conventional chemotherapy. Additionally, ADCs achieve enhanced therapeutic efficacy by concentrating their effects at the tumor site, which may result in improved response rates and overall survival outcomes for patients (17). The prolonged circulation time afforded by the large size of antibodies also contributes to their effectiveness by providing sustained exposure of tumor cells to the cytotoxic drug. Thus, ADCs represent a promising advancement in oncology, offering new hope for patients with difficult-to-treat malignancies such as ovarian cancer. Their unique mechanism and significant advantages position them as an important component of the evolving landscape of precision medicine (18).

#### 2.3.3. Investigational Antibody-Drug Conjugates and Clinical Trials in Ovarian Cancer

##### 2.3.3.1. Anti-human Epidermal Growth Factor Receptor 2-Based Antibody-Drug Conjugates

Human epidermal growth factor receptor 2 (HER2) is a protein that can be overexpressed in ovarian cancer. Anti-HER2-based ADCs represent a promising therapeutic strategy for treating HER2-positive ovarian cancer. These ADCs combine the specificity of anti-HER2 antibodies with potent cytotoxic agents, enabling targeted delivery to tumor cells while minimizing damage to healthy tissues. Several notable ADCs targeting HER2 are currently under investigation or have demonstrated potential in clinical trials (19).

Ado-trastuzumab emtansine is an ADC that combines trastuzumab, a humanized anti-HER2 antibody, with the cytotoxic agent emtansine (DM1), which disrupts microtubule function. T-DM1 has demonstrated significant efficacy in HER2-positive breast cancer and is being explored for its therapeutic potential in ovarian cancer. Early studies suggest that T-DM1 may provide a treatment option for patients with HER2-overexpressing tumors (20).

The T-DXd is a novel HER2-directed ADC consisting of trastuzumab linked to a topoisomerase I inhibitor, DXd. This ADC has shown promising results in various solid tumors, including ovarian cancer. Clinical trials have reported response rates of 54.5% in HER2-high expressing tumors and 70% in HER2-low expressing tumors, indicating its effectiveness across different levels of HER2 expression. The unique mechanism of action allows T-DXd to induce cell death not only in HER2-positive cells but also in adjacent cells, enhancing its therapeutic potential (21, 22).

Trastuzumab duocarmazine is another ADC under investigation for the treatment of ovarian cancer. Preclinical studies have shown that it exhibits potent antitumor activity against epithelial ovarian carcinoma cell lines, regardless of HER2 expression levels. Although clinical trials specifically exploring its efficacy in ovarian cancer are still pending, initial findings suggest it may be a valuable addition to the treatment landscape (23, 24).

BDC-1001 is an investigational ADC that consists of a trastuzumab backbone conjugated to a cytotoxic agent. Early-phase clinical trials are assessing its safety and efficacy in patients with HER2-positive tumors, including those with ovarian cancer. Preliminary results indicate promising antitumor activity, warranting further investigation (25).

Hertuzumab-vc-monomethyl auristatin E (MMAE) is an experimental anti-HER2 ADC that has shown potential in preclinical studies for treating HER2-positive ovarian cancer. This ADC combines a humanized antibody with MMAE, a potent microtubule-disrupting agent. Preclinical evaluations suggest significant cytotoxicity against HER2-positive tumor cells, indicating its potential as a therapeutic option (26).

##### 2.3.3.2. Anti-folate Receptor Alpha-based Antibody-Drug Conjugates

The ADCs targeting folate receptor alpha (FRα) have emerged as a promising therapeutic strategy for ovarian cancer, particularly due to the high expression of FRα in tumor tissues. Several ADCs targeting FRα are currently under investigation, demonstrating their potential in clinical settings.

The MIRV is the first FRα-targeting ADC approved by the United States Food and Drug Administration (FDA) for treating platinum-resistant ovarian cancer. Clinical trials have demonstrated that MIRV significantly improves overall response rates, achieving an objective response rate (ORR) of approximately 42% in patients with high FRα expression. The FDA granted accelerated approval for MIRV on November 14, 2022, based on its efficacy in patients who had received one to three prior systemic treatment regimens. Ongoing studies are evaluating the combination of MIRV with other agents such as bevacizumab (BEV), which has shown notable anti-tumor activity and durable responses in recurrent ovarian cancer (27, 28).

MORAb-202 is an investigational ADC currently undergoing phase I/II trials for various solid tumors, including ovarian and endometrial cancers. This ADC combines a humanized anti-FRα monoclonal antibody with a cytotoxic payload, aiming to exploit the high expression of FRα in tumors. Early-phase clinical data suggest that MORAb-202 may offer a viable treatment option for patients with FRα-positive malignancies (29).

STRO-002 is a novel homogeneous ADC designed to target FRα, currently being evaluated in clinical trials for ovarian and endometrial cancers. Preclinical studies have demonstrated significant tumor growth inhibition in FRα-expressing xenograft models. A phase I dose escalation trial (NCT03748186) is underway to assess its safety and efficacy in patients with relapsed ovarian cancer, with initial results indicating potent anti-tumor activity (30).

##### 2.3.3.3. Anti-trophoblast Cell Surface Antigen 2-Based Antibody-Drug Conjugates

The ADCs targeting trophoblast cell surface antigen 2 (TROP2) have emerged as a promising therapeutic option for various cancers, including ovarian cancer. Multiple studies report that TROP2 is frequently overexpressed in subsets of ovarian cancer, underscoring its potential as a therapeutic target; however, expression levels may vary across tumor subtypes and cohorts, reflecting the heterogeneity characteristic of this disease. Several ADCs targeting TROP2 are currently under investigation, highlighting their potential in clinical settings (31).

Sacituzumab govitecan is a TROP2-directed ADC that has received FDA approval for treating metastatic triple-negative breast cancer (32). It combines a humanized anti-TROP2 antibody with the cytotoxic agent SN-38, a topoisomerase I inhibitor. Clinical trials have demonstrated significant antitumor activity in various solid tumors, including ovarian cancer. In a phase II trial, sacituzumab govitecan showed an ORR of 30.4% in patients with TROP2-expressing advanced solid tumors, indicating its potential effectiveness in this patient population (33).

Sacituzumab tirumotecan (SKB-264) is an investigational ADC designed to target TROP2 and is currently undergoing clinical evaluation. Early-phase studies have shown encouraging results regarding its safety and efficacy in patients with advanced solid tumors, including ovarian cancer. Ongoing clinical trials aim to assess the therapeutic potential of SKB-264 and its ability to provide meaningful responses in TROP2-positive malignancies (34).

PF-06664178 is another TROP2-targeting ADC that is currently in clinical trials. This agent combines an anti-TROP2 antibody with a potent cytotoxic payload, aiming to exploit high TROP2 expression in tumor cells. Preliminary results from early-phase studies suggest that PF-06664178 may offer a viable treatment option for patients with TROP2-overexpressing tumors, although further data are needed to establish its clinical efficacy (35).

The Dato-DXd is an investigational ADC that targets TROP2 and has exhibited promising preclinical and early clinical activity against TROP2-overexpressing ovarian cancers. In a phase II multicenter study, Dato-DXd achieved an ORR of 42.9% in patients with recurrent ovarian cancer who had progressed on prior platinum-based chemotherapy. These promising results support the continued development of Dato-DXd as a potential therapeutic option for this patient population (36, 37).

Further ADCs targeting TROP2 are also under investigation, including agents employing different cytotoxic agents or linker technologies to enhance therapeutic efficacy while minimizing toxicity.

##### 2.3.3.4. Anti-mesothelin-Based Antibody-Drug Conjugates

Anti-mesothelin ADCs represent an emerging therapeutic approach for treating mesothelin-expressing tumors, including ovarian cancer. Mesothelin is a glycoprotein overexpressed in various malignancies, making it an attractive target for therapies designed to deliver cytotoxic agents directly to tumor cells while minimizing damage to normal tissues (38). Several notable ADCs targeting mesothelin are currently under investigation.

Anetumab ravtansine (BAY 94-9343) is a novel ADC composed of a fully human anti-mesothelin antibody conjugated to the maytansinoid DM4 via a disulfide-containing linker. This ADC has demonstrated strong antitumor activity in preclinical models of ovarian cancer and other mesothelin-expressing tumors. In clinical trials, BAY 94-9343 has shown promising efficacy, particularly in patients with advanced solid tumors, including ovarian cancer. Its ability to induce a bystander effect enhances its therapeutic potential, enabling effective targeting of heterogeneous tumor populations (39).

BMS-986148 is an investigational ADC targeting mesothelin, engineered to deliver a cytotoxic payload specifically to mesothelin-expressing cells. This ADC combines a fully human IgG1 anti-mesothelin monoclonal antibody with tubulysin, a potent microtubule-disrupting agent. Early-phase clinical trials have demonstrated its safety and preliminary efficacy in patients with mesothelin-expressing tumors. BMS-986148 is currently being evaluated in combination with nivolumab, an anti-PD-1 therapy, to enhance antitumor activity through complementary mechanisms (40).

DMOT4039A is another anti-mesothelin ADC under investigation for its potential in treating ovarian cancer and other solid tumors. This ADC utilizes a humanized anti-mesothelin antibody linked to a cytotoxic agent, enabling selective targeting and killing of mesothelin-positive cancer cells. Early clinical studies have reported encouraging results, suggesting DMOT4039A may provide a viable treatment option for patients with advanced mesothelin-expressing malignancies (41).

RC88 is an investigational ADC that targets mesothelin and is currently being evaluated in clinical trials for various cancers, including ovarian cancer. Preliminary data suggest that RC88 may exhibit significant antitumor activity against mesothelin-positive tumors, though further studies are needed to establish its clinical efficacy (42).

Additional ADCs targeting mesothelin are also in development, including those employing different cytotoxic agents or linker technologies to enhance therapeutic efficacy while minimizing toxicity.

##### 2.3.3.5. Anti-sodium-Dependent Phosphate Transporter 2B-Based Antibody-Drug Conjugates

Anti-NaPi2B ADCs are a promising therapeutic approach for treating various cancers, including ovarian cancer, owing to the overexpression of NaPi2B in tumor tissues. NaPi2B is a sodium-dependent phosphate transporter highly expressed in several malignancies, making it an attractive target for ADCs designed to deliver cytotoxic agents directly to cancer cells. Several notable ADCs targeting NaPi2B are currently under investigation (43).

Lifastuzumab vedotin (DNIB0600A) is a humanized anti-NaPi2B monoclonal antibody conjugated to MMAE, a potent microtubule inhibitor. In early-phase clinical trials, DNIB0600A has shown promising activity in patients with platinum-resistant ovarian cancer (44). A phase II study demonstrated that patients receiving lifastuzumab vedotin had an ORR of 34%, compared to 15% for those treated with standard pegylated liposomal doxorubicin (PLD). Although improvements in progression-free survival (PFS) were not statistically significant, the response rates highlight lifastuzumab vedotin’s potential as a therapeutic option for NaPi2B-positive tumors (45).

Upifitamab rilsodotin (ABBV-181) is an investigational ADC targeting NaPi2B, designed to deliver a cytotoxic agent specifically to NaPi2B-expressing cells. Early clinical studies have indicated its safety and preliminary efficacy in various solid tumors, including ovarian cancer. Ongoing trials aim to establish its therapeutic potential and optimize treatment strategies for patients with NaPi2B-positive malignancies (46, 47).

ZW220 is an innovative ADC explicitly designed to target NaPi2B, which is significantly overexpressed in ovarian cancer, particularly in serous adenocarcinoma. ZW220 combines a fully humanized IgG1 antibody that selectively binds to NaPi2B with a potent cytotoxic agent, ZD06519, a topoisomerase I inhibitor. Preclinical studies have demonstrated that ZW220 exhibits compelling antitumor efficacy in various ovarian cancer models (48).

##### 2.3.3.6. Anti-mucin 16-Based Antibody-Drug Conjugates

Anti-MUC16 ADCs are emerging as a promising therapeutic strategy for ovarian cancer, particularly due to the overexpression of MUC16 (also known as CA125) in many ovarian tumors. MUC16 is a tumor-associated antigen expressed on the surface of cancer cells, making it a compelling target for ADCs designed to deliver potent cytotoxic agents directly to these tumors. Several notable ADCs targeting MUC16 are currently under investigation (49, 50).

Sofituzumab vedotin (DMUC5754A) is an investigational ADC targeting MUC16, combining a humanized anti-MUC16 monoclonal antibody with MMAE. This ADC has been evaluated in phase I clinical trials in patients with advanced recurrent platinum-resistant ovarian cancer and has shown evidence of antitumor activity, particularly in patients whose tumors express high levels of MUC16 (51).

DMUC4064A is an anti-MUC16 THIOMAB^™^-drug conjugate that utilizes a novel approach for achieving a more homogeneous payload distribution than traditional ADCs. This humanized anti-MUC16 monoclonal antibody is linked to MMAE, a potent microtubule-disrupting agent. In a phase I study, DMUC4064A exhibited a tolerable safety profile and preliminary antitumor activity in approximately 25% of patients with platinum-resistant ovarian cancer, indicating its potential as a viable treatment option (52).

Abagovomab is an anti-MUC16 monoclonal antibody conjugated via a maleimidocaproyl (MC) linker to monomethyl auristatin F (MMAF), another cytotoxic agent. This ADC aims to exploit the overexpression of MUC16 in certain cancers by delivering MMAF directly into cancer cells through receptor-mediated endocytosis, disrupting microtubule dynamics and inducing cell death (53, 54).

##### 2.3.3.7. Anti-anexelekto-Based Antibody-Drug Conjugates

Anti-AXL ADCs are an innovative therapeutic strategy aimed at targeting the AXL receptor. AXL expression is elevated in specific ovarian cancer subtypes and plays a role in tumor progression and therapy resistance, although expression levels may vary depending on tumor context. AXL is a receptor tyrosine kinase with a critical role in tumor progression, metastasis, and resistance to chemotherapy. By targeting AXL, ADCs can potentially deliver cytotoxic agents directly to tumor cells while minimizing damage to normal tissues (55-57).

Enapotamab vedotin is an investigational anti-AXL ADC that combines a monoclonal antibody targeting AXL with the cytotoxic agent MMAE. This ADC is designed to exploit the overexpression of AXL in tumor cells, allowing for selective targeting and internalization. Early clinical trials have demonstrated promise in patients with advanced malignancies, including ovarian cancer, indicating its potential as a treatment option for AXL-positive tumors (58-60).

Mipasetamab uzoptirine is another promising ADC targeting AXL, currently under clinical evaluation for various cancers. This ADC is designed to exploit the expression profile of AXL in tumor cells to deliver potent cytotoxic agents selectively. Early studies suggest potential antitumor activity, although additional clinical data are needed to establish its efficacy (55, 61, 62).

##### 2.3.3.8. Anti-protein Tyrosine Kinase 7-Based Antibody-Drug Conjugates

Anti-PTK7 ADCs represent an emerging therapeutic strategy targeting PTK7, a receptor implicated in cancer progression and treatment resistance. PTK7 is often overexpressed in various cancers, including ovarian cancer, making it a compelling target for ADCs delivering cytotoxic agents specifically to cancer cells (63, 64)

Cofetuzumab pelidotin is an anti-PTK7 ADC that combines a humanized monoclonal antibody targeting PTK7 with the potent cytotoxic agent pelidotin, a derivative of the auristatin class of microtubule inhibitors. Preclinical studies have demonstrated that cofetuzumab pelidotin effectively reduces tumor growth in PTK7-expressing tumors, including those derived from ovarian cancer (65, 66).

##### 2.3.3.9. Anti-ephrin-A4-Based Antibody-Drug Conjugates

Anti-EFNA4 ADCs represent an innovative therapeutic strategy targeting EFNA4, a protein often overexpressed in various cancers, including ovarian cancer. EFNA4 has been associated with aggressive tumor behavior and is expressed in tumor-initiating cells (TICs), making it a compelling target for ADCs designed for targeted cytotoxic delivery (67, 68).

PF-06647263 is a notable anti-EFNA4 ADC consisting of a humanized anti-EFNA4 monoclonal antibody linked to the potent cytotoxic agent calicheamicin. This ADC has demonstrated significant antitumor activity in preclinical studies, particularly in patient-derived xenograft (PDX) models of ovarian cancer. In these models, PF-06647263 achieved sustained tumor regressions and reduced the frequency of TICs, indicating its potential to induce durable clinical responses (69). PF-06647263 has been evaluated in a first-in-human phase I study (NCT02078752), which assessed safety and efficacy in patients with advanced solid tumors, including ovarian cancer. The study reported manageable safety profiles with mild to moderate adverse effects and provided preliminary evidence of antitumor activity, with some patients achieving stable disease (70). Ongoing research may yield additional candidates targeting EFNA4 or related ephrin family members. The focus on EFNA4 as a therapeutic target is supported by its elevated expression in aggressive tumor cell populations across various cancers.

##### 2.3.3.10. Anti-dipeptidase 3-Based Antibody-Drug Conjugates

Anti-DPEP3 ADCs represent an innovative therapeutic approach targeting DPEP3, a membrane-bound glycoprotein associated with TICs in ovarian cancer. DPEP3 is frequently overexpressed in high-grade serous ovarian carcinoma (HGSC), providing a promising target for ADCs designed to deliver cytotoxic agents specifically to these cancer cells (71).

Tamrintamab pamozirine, also known as SC-003, is a notable anti-DPEP3 ADC that combines a humanized monoclonal antibody targeting DPEP3 with the potent cytotoxic agent pyrrolobenzodiazepine (PBD). SC-003 has demonstrated significant preclinical efficacy in PDX models of ovarian cancer, including those that are platinum-resistant. In these studies, a single dose of SC-003 was sufficient to induce tumor regression, highlighting its potential as a therapeutic option for patients with advanced DPEP3-positive ovarian cancer (72). A first-in-human phase 1a/1b study (NCT02539719) evaluated the safety, tolerability, pharmacokinetics, and preliminary antitumor activity of SC-003 in patients with platinum-resistant or refractory ovarian cancer. The trial enrolled seventy-four patients and reported manageable safety profiles with mild to moderate adverse effects. Although the overall response rate was low (4%), post hoc analyses suggested that higher DPEP3 expression correlated with improved response rates, underscoring the importance of patient selection based on biomarker expression (73).

##### 2.3.3.11. Anti-CD116-Based Antibody-Drug Conjugates

Anti-CD116 ADCs represent an emerging therapeutic strategy targeting CD116, also known as the human receptor for granulocyte-macrophage colony-stimulating factor (GM-CSF). CD116 is involved in the regulation of hematopoiesis and immune responses and is expressed in various cancers, including ovarian cancer, making it a compelling target for ADC-mediated cytotoxic delivery (74, 75).

Praluzatamab ravtansine (also known as ABBV-181) is a notable anti-CD166 ADC that combines a monoclonal antibody targeting CD166 with the potent cytotoxic agent DM4 (ravtansine). This ADC has demonstrated promising preclinical efficacy and is currently undergoing clinical evaluation in patients with advanced solid tumors, including ovarian cancer. The mechanism of action involves binding to CD116 on the surface of cancer cells, leading to internalization of the conjugate and subsequent release of the cytotoxic agent, inducing cell death (76).

Praluzatamab ravtansine is currently being evaluated in clinical trials for its safety and efficacy in patients with advanced ovarian cancer. Early-phase studies have reported manageable safety profiles and preliminary evidence of antitumor activity, indicating its potential as a treatment option for patients with CD116-positive tumors (77).

## 3. Results

The development of ADCs for ovarian cancer has been driven by the identification of specific tumor-associated antigens that are overexpressed on cancer cells (Table 1). Targeting these antigens allows for more precise therapeutic interventions, enhancing the efficacy of treatment while minimizing toxicity to normal tissues. As ovarian cancer frequently presents at advanced stages and is associated with a high rate of recurrence, the need for effective targeted therapies has never been more critical (78). The expansion of potential therapeutic targets is expected to lead to more effective strategies, ultimately improving outcomes for patients facing this challenging disease (79).

The landscape of ADCs in ovarian cancer is rapidly evolving, with numerous clinical trials currently underway to assess their efficacy and safety in various clinical settings (Table 2). Clinical development in this field has progressed swiftly, with several agents now in late-stage trials or already approved. Notably, MIRV, which targets FRα, has become the first United States FDA-approved ADC specifically for ovarian cancer, representing a significant milestone in the evolution of targeted therapy for this indication. Beyond FRα-targeted therapies, ADCs directed against HER2, TROP2, mesothelin, and NaPi2B have demonstrated promising clinical activity in ongoing trials. Early-phase trials have shown promising antitumor activity, suggesting that these novel therapies could have a meaningful impact on treatment outcomes for patients with advanced disease (80).

The ongoing exploration of ADCs in clinical trials reflects a broader trend toward personalized medicine in oncology (81). The ADCs have emerged as a transformative approach in the treatment of ovarian cancer, particularly given the high unmet need for effective therapies in this patient population. With the ability to target specific tumor-associated antigens, ADCs offer a promising strategy to deliver potent cytotoxic agents directly to cancer cells while minimizing systemic toxicity (82).

The landscape of ADC development is rapidly expanding, with over thirty ADCs targeting various biomarkers currently under investigation for ovarian cancer. This includes targets such as mesothelin and cadherin 6; the identification of these targets reflects an enhanced understanding of tumor biology and supports the need for personalized treatment strategies that address the unique characteristics of each patient's cancer (83). The composition and design of ADCs targeting ovarian cancer antigens have been summarized in Table 3. 

The therapeutic advancement of ADCs is reflected in market growth projections. The global ovarian cancer market, including ADCs, was valued at approximately USD 2.3 billion in 2024 and is projected to grow to USD 5.5 billion by 2033, at a compound annual growth rate (CAGR) of 9.6%. The ADC segment is among the fastest growing, with a CAGR exceeding 13%, driven by innovations in antibody engineering, linker chemistry, and payload design, as well as rising ovarian cancer incidence and increased demand for targeted therapies. This growth underscores ADCs as key biopharmaceuticals poised to substantially improve patient outcomes.

## 4. Discussion

The ADCs have emerged as a promising therapeutic strategy for ovarian cancer, particularly for patients with limited available treatment options. However, several challenges and limitations hinder their full effectiveness and wider application. This discussion focuses on two key areas: Resistance mechanisms and toxicity or safety concerns.

### 4.1. Resistance Mechanisms

Resistance to ADCs in ovarian cancer can arise from several factors that may compromise therapeutic efficacy.

#### 4.1.1. Antigen Downregulation

One of the primary mechanisms of resistance is the downregulation of the target antigen on tumor cells. Chronic exposure to an ADC can lead to decreased expression of the antigen, reducing binding affinity and subsequent internalization. For example, studies involving ADCs targeting FRα have shown that persistent treatment may diminish FRα levels on cancer cells, thereby impairing the ADC’s ability to deliver its cytotoxic payload effectively (10).

#### 4.1.2. Altered Drug Transport

The overexpression of drug efflux transporters, such as P-glycoprotein (MDR1) and multidrug resistance-associated proteins (MRP1), can lead to reduced intracellular concentrations of the cytotoxic drug delivered by the ADC. This efflux mechanism diminishes the drug's effectiveness, allowing cancer cells to survive despite ADC treatment (84).

#### 4.1.3. Changes in Intracellular Processing

**Figure 3.**
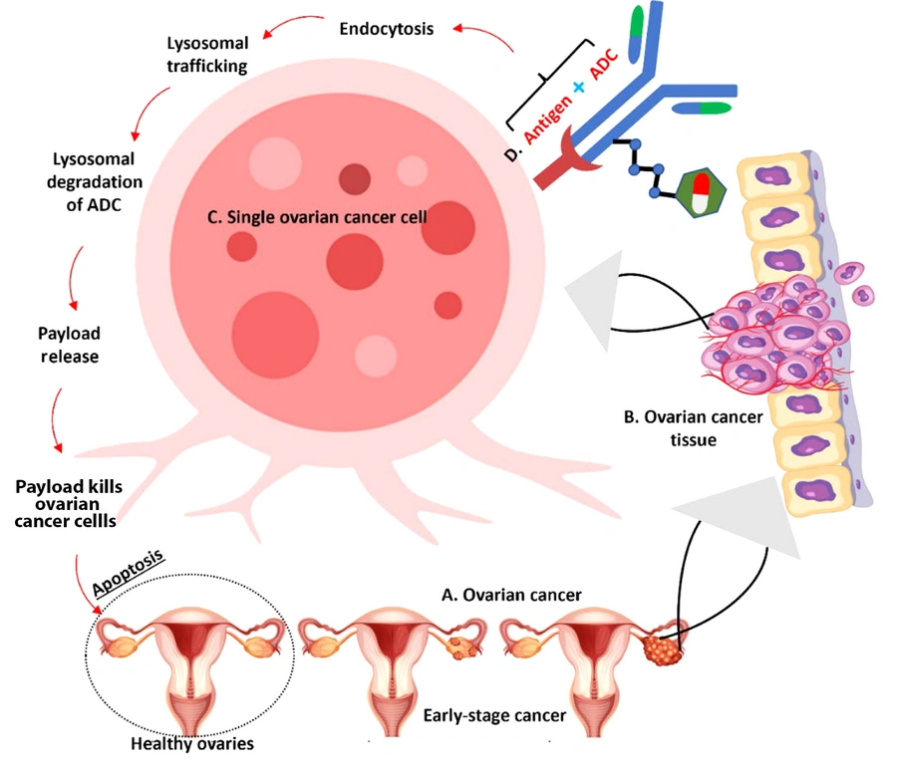
Molecular Mechanism of action of antibody-drug conjugates (ADCs) in targeting ovarian cancer cells – the illustration demonstrates the progression from healthy to cancerous ovarian tissue and the mechanism of action of ADCs: A, ovarian cancer development showing progression from healthy ovaries to early-stage cancer; B, ovarian cancer tissue showing tumor formation; C, enlarged view of a single ovarian cancer cell demonstrating the detailed mechanism; D, ADC binding to target antigen on the cell surface.

Resistance can also develop from alterations in the cellular processing of ADCs. Changes in endosomal trafficking or lysosomal degradation pathways may affect the release of the cytotoxic agent from the ADC, limiting its capacity to induce cell death (Figure 3) (85).

#### 4.1.4. Tumor Microenvironment

The tumor microenvironment can influence ADC efficacy by creating physical barriers that impede drug delivery. Factors such as increased interstitial pressure or a dense extracellular matrix may limit ADC diffusion from the bloodstream to tumor cells, reducing treatment effectiveness (86).

#### 4.1.5. Cancer Stem Cells

The presence of CSCs within ovarian tumors presents a significant challenge, as these cells often exhibit inherent resistance to conventional therapies and may evade ADC-mediated cell death. CSCs may express different surface markers than bulk tumor cells, complicating targeted therapeutic approaches (87, 88).

### 4.2. Toxicity and Safety Concerns

While ADCs are designed to minimize off-target toxicity by selectively targeting cancer cells, adverse effects remain a clinical concern.

#### 4.2.1. Adverse Effects

Common side effects associated with ADCs include fatigue, nausea, vomiting, and hematological toxicities such as neutropenia and thrombocytopenia. These effects can result from both the cytotoxic payload and immune-mediated reactions related to antibody infusion. For example, MIRV has been associated with ocular toxicity, necessitating careful monitoring during treatment (89).

#### 4.2.2. Off-target Toxicity

Despite their targeted action, some ADCs may bind to non-cancerous tissues that express low levels of the target antigen, resulting in unintended toxicity. For instance, targeting FRα may also affect normal tissues with low receptor expression.

#### 4.2.3. Immunogenicity

The potential for immunogenic reactions against the antibody component of ADCs can lead to hypersensitivity reactions or diminished therapeutic efficacy over time due to the development of anti-drug antibodies (ADAs). These immune responses may neutralize the therapeutic effect or cause allergic reactions (90, 91).

#### 4.2.4. Long-term Effects

The long-term safety profile of novel ADCs remains uncertain, as many are still undergoing clinical evaluation. Continuous monitoring is required to identify any late-onset toxicities that may appear after prolonged use (92).

### 4.3. Conclusions

In summary, this review highlights significant advancements and ongoing challenges associated with ADCs in the treatment of ovarian cancer. Key findings include the identification of various ADCs targeting specific tumor-associated antigens — such as FRα, HER2, TROP2, and DPEP3 — which have shown promising efficacy in clinical trials. Additionally, the review discusses the mechanisms of resistance that may limit the effectiveness of ADCs and the potential adverse effects that can arise from their use.

### 4.4. Future Directions and Perspectives

The field of ADC development is rapidly evolving, with emerging technologies and innovations aimed at enhancing their design and efficacy. Next-generation ADCs are being engineered with improved linkers that provide greater stability and enable more efficient drug release mechanisms within tumor cells. Recent advances include cleavable linkers responsive to tumor-specific stimuli (such as low pH, proteases, or redox conditions) and non-cleavable linkers that rely on lysosomal degradation. Innovations in site-specific conjugation methods, such as engineered cysteine residues or enzymatic ligation, offer improved product homogeneity and controlled drug-to-antibody ratios (DAR), leading to reduced off-target toxicity. Emerging linker chemistries, including hydrazone, disulfide, and click-chemistry-based linkers, allow for tunable release rates and enhanced pharmacokinetics. Collectively, these advances promise more effective and safer ADCs, supporting their growing role in ovarian cancer therapy.

Additionally, advances in antibody engineering — such as the development of bispecific antibodies or novel targeting strategies — are being investigated to expand the spectrum of tumors that can be effectively targeted (11, 93, 94). These innovations hold the potential to overcome current limitations and introduce new therapeutic options for patients with ovarian cancer. As research continues to progress, these developments may fundamentally reshape the landscape of ovarian cancer therapy, offering hope for improved survival and quality of life.
